# Supplementary material for: HLA-A*01:01 allele diminishing in COVID-19 patients population associated with non-structural epitope abundance in CD8+ T-cell repertoire
Source: PeerJ. 2023 Jan 18;11:e14707. doi: 10.7717/peerj.14707 (PMC9864130; doi:10.7717/peerj.14707)
Supplement: Supplemental Information 2 — OR (p-value). [file peerj-11-14707-s002.docx]

**Supplementary Table S2:**

**The relationship of comorbidities to the risk of death from COVID-19.** OR (p-value).

|  | **Wave 1** | **Wave 3** |
| --- | --- | --- |
| Obstructive pulmonary disease | 0 (1) | 0 (0,6) |
| Obesity | 0 (1) | 2.3 (0.24) |
| Heart diseases | **13 (5.2e-05)** | 2.5 (0.12) |
| Hypertension | **3.5 (0.045)** | 1.3 (0.6) |
| Neoplasma | **inf (0.002)** | **5 (0.049)** |
