# Supplementary material for: HLA-A*01:01 allele diminishing in COVID-19 patients population associated with non-structural epitope abundance in CD8+ T-cell repertoire
Source: PeerJ. 2023 Jan 18;11:e14707. doi: 10.7717/peerj.14707 (PMC9864130; doi:10.7717/peerj.14707)
Supplement: Supplemental Information 4 [file peerj-11-14707-s004.docx]

**Supplementary Table S4:**

**A panel of ORF1ab epitopes restricted to HLA-A*01:01 and HLA-A*02:01.**

| **Peptide** | **Location** | **Predicted binding affinity to** | |
| --- | --- | --- | --- |
|  |  | **HLA-A*01:01** | **HLA-A*02:01** |
| CTDDNALAYY | Non-structural proteins | 3 | 19896 |
| TTDPSFLGRY | Non-structural proteins | 5 | 27903 |
| DTDFVNEFY | Non-structural proteins | 6 | 33483 |
| GTDLEGNFY | Non-structural proteins | 9 | 33917 |
| PTDNYITTY | Non-structural proteins | 12 | 35372 |
| NTCDGTTFTY | Non-structural proteins | 14 | 25394 |
| HTTDPSFLGRY | Non-structural proteins | 42 | 33468 |
| YLDAYNMMI | Non-structural proteins | 221 | 2 |
| FTYASALWEI | Non-structural proteins | 5189 | 17 |
| FLLNKEMYL | Non-structural proteins | 13747 | 2 |
| YLFDESGEFKL | Non-structural proteins | 18838 | 6 |
| ALWEIQQVV | Non-structural proteins | 27002 | 5 |
| NMLRIMASL | Non-structural proteins | 29822 | 46 |
| RQLLFVVEV | Non-structural proteins | 31003 | 17 |
| KLWAQCVQL | Non-structural proteins | 33067 | 8 |
