# Supplementary figures and images for: HLA-A*01:01 allele diminishing in COVID-19 patients population associated with non-structural epitope abundance in CD8+ T-cell repertoire
Source: PeerJ. 2023 Jan 18;11:e14707. doi: 10.7717/peerj.14707 (PMC9864130; doi:10.7717/peerj.14707)

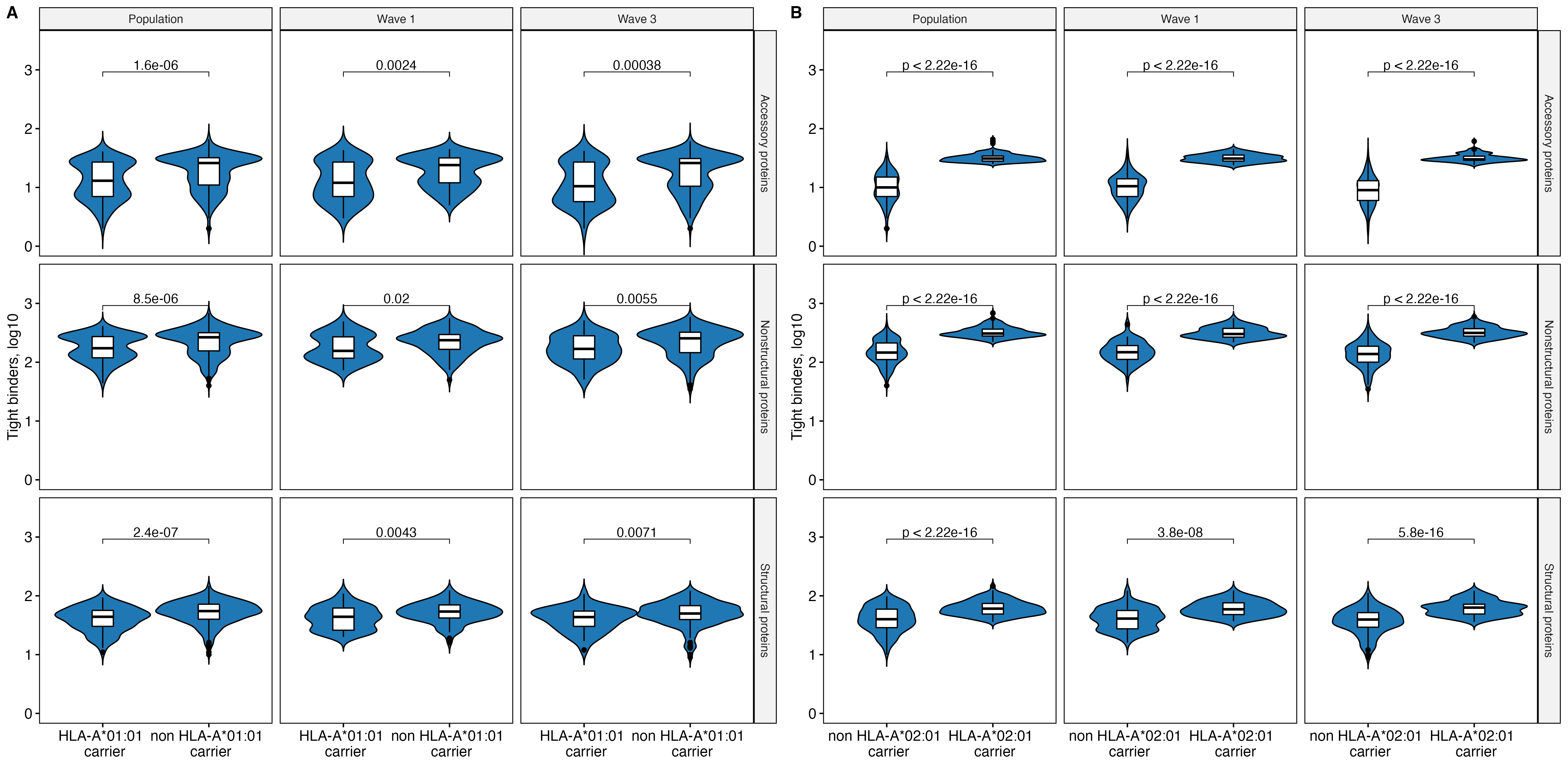

Supplement: Supplemental Information 6 — (A) Comparison of HLA-A*01:01 vs non HLA-A*01:01 carriers. (B) Comparison of HLA-A*02:01 vs non HLA-A*02:01 carriers. [file peerj-11-14707-s006.png]

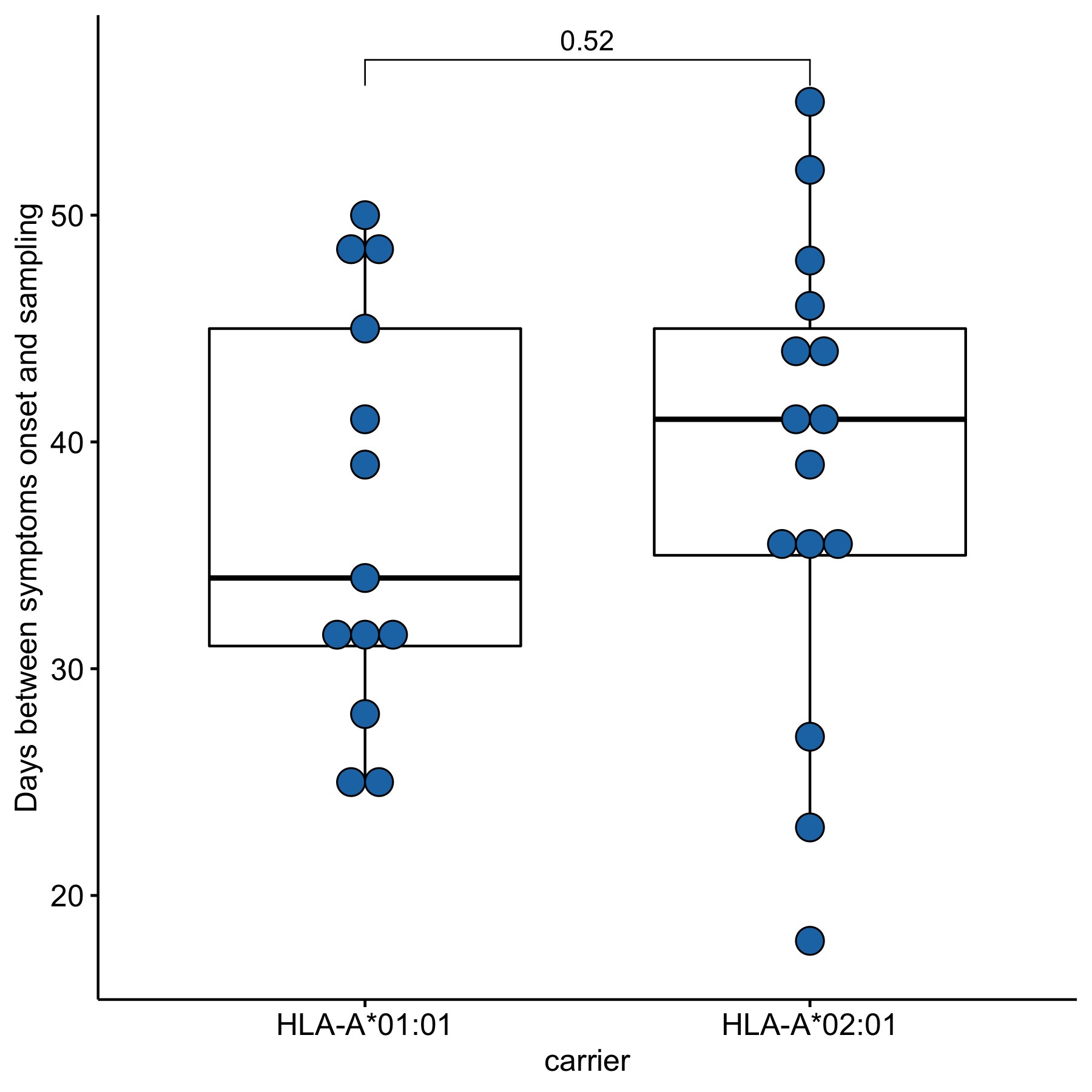

Supplement: Supplemental Information 7 [file peerj-11-14707-s007.png]
